# Insights into nutrition, flavor and edible quality changes of golden pomfret (*Trachinotus ovatus*) fillets prepared by different cooking methods

**DOI:** 10.3389/fnut.2023.1227928

**Published:** 2023-07-07

**Authors:** Tian Xiong, Xin Mei, Yanyan Wu, Lan Wang, Jianbin Shi, Yong Sui, Sha Cai, Fang Cai, Xueling Chen, Chuanhui Fan

**Affiliations:** ^1^Key Laboratory of Agricultural Products Cold Chain Logistics, Ministry of Agriculture and Rural Affairs of the P.R. China, Institute of Agro-Product Processing and Nuclear Agricultural Technology, Hubei Academy of Agricultural Sciences, Wuhan, China; ^2^Key Laboratory of Aquatic Product Processing, Ministry of Agriculture and Rural Affairs of the P.R. China, South China Sea Fisheries Research Institute, Chinese Academy of Fishery Science, Guangzhou, China

**Keywords:** golden pomfret (*Trachinotus ovatus*), thermal processing, nutrition, flavor, texture, sensory evaluation

## Abstract

**Introduction:**

In recent years, Asia has seen an increase in demand for golden pomfret (*Trachinotus ovatus*). Especially in instant (ready-to-eat) and prepared (ready-to-cock) food processing industry. Thermal processing is a vital part of food processing. However, no comprehensive analysis has been reported on its flavor, nutrition and edible quality changes during the key thermal processing.

**Methods:**

Accordingly, in this study, we evaluated the effects of different cooking methods (steaming, frying, microwaving and baking) on the color, texture, cooking loss, nutrition composition, volatile flavor substances and other indicators of golden pomfret filets.

**Results and Discussion:**

The results showed that the steamed samples (SS) had the lowest cooking loss and fat content, the highest moisture content, complete appearance and the lowest levels of hardness and chewiness. Fried samples (FS) had a notable difference in fatty acid composition. The content of unsaturated fatty acids (UFAs) increased significantly, while the relative content of eicosapentaenoic acid (EPA) + docosahexaenoic acid (DHA) decreased from 7.88 to 1.42%, lower than other groups. The essential amino acid index (EAAI) of microwaved samples (MS) was 94.89, which was higher than other groups. Baked samples (BS) had the highest relative content of umami amino acids (UAAs) and sweet amino acids (SAAs), which was 8.08 mg/100 mg and 5.19 mg/100 mg, respectively. Hexanal and nonanal were detected in control samples (CK), SS, FS, MS and BS. While pyrazine compounds were detected only in FS and BS. Steaming and microwaving treatment of golden pomfret resulted in better nutritional preservation, which was more conducive to human health. Frying and baking treatment of golden pomfret had better taste and flavor and higher sensory scores. The nutrition, flavor and edible quality of golden pomfret under different cooking methods were related and interactive. Cooking loss and fat content can be used as simple evaluation indicators to compare the overall quality of different cooking methods. This study provides a reference for the thermal processing technology and industrial production of golden pomfret.

## 1. Introduction

As a warm water fish, golden pomfret (*Trachinotus ovatus*) is mainly distributed in tropical and temperate regions (1). It has great development potential and is widely cultivated as a new type of introduced fish species in China, Singapore and Malaysia. Golden pomfret is nutritious, especially rich in protein, lipids and moisture, which is prone to deterioration (2). There has been a lot of research in the past focusing on maintaining the storage quality and extending the shelf life of fresh and frozen fish, but such unprocessed fish has low added value and usually brings fewer returns (3, 4). Recently, some research developed innovative approaches to fermented fish to enhance the nutrition and flavor and extend the storage period to make it highly profitable (5, 6). However, this method is usually time-consuming, its flavor is unstable and its safety cannot be guaranteed (7). Usually, thermal treatment is used to develop microbial-safe fish products with extended shelf life. The thermal processing of golden pomfret into instant(ready-to-eat) and prepared(ready-to-cock) food can ensure its stable product quality and meet the consumer demand for convenience and speed.

Thermal processing brings both positive and negative changes to different foods processed at different heat processing intensities (8, 9). For plant-based foods, the functional active ingredients are destroyed and starch pasting brings nutritional, color and flavor changes (10, 11). While, for meat foods, more protein denaturation and juice loss cause meat quality, color and flavor changes (12, 13). Published studies on the thermal processing of golden pomfret have focused on the effects of drying methods and drying temperatures on flavor (14, 15). In addition, the above thermal processing was in the low-temperature heating region (40–100°C). However, no studies have been conducted on the effects of different processing methods on golden pomfret in the high-temperature heating region (100–280°C). The reported studies on the effects of different cooking methods of aquatic products have focused on nutrition, digestive properties and flavor (16–19). There is a lack of research that can provide comprehensive information to guide the production and consumption of instant and prepared golden pomfret products in the market.

Therefore, to find the scientific and suitable thermal processing methods of golden pomfret in the high-temperature heating region, the differences and correlation in flavor, nutrition and edible quality of fresh, steamed, fried, microwaved and baked golden pomfret were comprehensively compared and analyzed. This study provides a reference for thermal processing processes and industrial production of golden pomfret. In addition, it will help to promote diversified deep processing of aquatic products and the flourishing of aquaculture and food processing industries.

## 2. Materials and methods

### 2.1. Materials

A total of 20 live golden pomfrets, with lengths of 28.55 ± 2.05 cm and weights of 452.31 ± 45.81 g, were purchased from a local fish market (Guangzhou, China). They were kept in a plastic container with sea water and transported to the laboratory within 30 min. The transportation temperature maintained at 20–25°C. Duo Li sunflower oil, 2.5 L, was purchased from Guangzhou China Resources Vanguard Supermarket. Chemicals, such as sodium hydroxide, petroleum ether and n-hexane, were obtained from Sinopharm Chemical Reagent Co., Ltd. (Shanghai, China).

### 2.2. Treatment of samples

The samples were processed with reference to the method of Wang et al. (12) and the method was modified according to the pre-experiment. Fresh golden pomfrets were cleaned and the back muscles of the fish were sliced and trimmed into filets of 2 cm × 2 cm × 1 cm (length × width × height) as samples to be tested. Samples were randomly divided into 5 homogenous groups of 20 filets each. The filets without cooking were the control samples (CK). For steaming cooking, the samples were performed at approximately 100°C (steaming temperature) for 2.5 min (SS). Fried samples were done with sunflower oil in a frying pan of 1.5 L capacity at a temperature of approximately 180°C for 2.5 min (FS). Microwaved samples (MS) were conducted at 500 W for 2.5 min in the micro-wave mode of a Lightwave-microwave oven (Galanz G80F23, China). Baked samples (BS) were prepared by the barbecue (light-wave) mode of a Lightwave-microwave oven (Galanz G80F23, China) for 2.5 min.

All cooking experiments were performed in triplicate, and all samples were drained on the surface with filter paper, and then packed in vacuum-sealed aluminum bags and kept in a fridge (−20°C) until further analyzes, which took less than 5 days. Samples used to analyze nutrients and flavors were manually de-boned and ground in a kitchen blender to ensure homogeneity and representative samples for analysis.

### 2.3. Determination of color

The changes in color between samples were measured by an automatic colorimeter (CR-410 Automatic Chromometer, Konica Minolta, Japan) at room temperature. The colorimeter was calibrated by standard whiteboard and blackboard, and the illuminant was set at D65. Each sample was measured 6 times, and nine sites on the pileus surface were randomly selected to record the value of the lightness (L^*^), red-green value (a^*^) and yellow-blue value (b^*^). Then the whiteness (*W*), chroma (C^*^), hue angle (*H*) and total color difference (*△E*) was calculated according to the following formula (20, 21):


$$W=100-\sqrt{{\left(100-L^{*}\right)}^{2}+a^{*2}+b^{*2}}$$

$$C^{*}=\sqrt{{\left(a^{*}\right)}^{2}+{\left(b^{*}\right)}^{2}}$$

$$\Delta E^{*}=\sqrt{{\left(\Delta L^{*}\right)}^{2}+{\left(\Delta a^{*}\right)}^{2}+{\left(\Delta b^{*}\right)}^{2}}$$

$$H^{*}=\tan^{-1}\left(b^{*}/a^{*}\right)$$

### 2.4. Texture profile

The samples were examined by a CT3-4500 Texture Analyzer (Brookfield Engineering Laboratories, United States) equipped with Texture Pro CT software as previously reported (22). The test conditions were two consecutive cycles of 30% compression with a trigger point of 5 g. A cylindrical probe, 4 mm in diameter, was used to analyze the texture profiles. The measured parameters were hardness, springiness, cohesiveness and chewiness. The reported data are the averages of 6–8 times repeat test of each sample.

### 2.5. Cooking loss

Cooking loss was calculated from the difference in sample mass before and after cooking. Before cooking, the surfaces of fish slices were wiped with filter paper and then weighed. After cooking, they were cooled to room temperature. The surfaces of the fish filets were blotted with filter papers and weighed again. Each sample was repeated 5 times.

### 2.6. Proximate composition

Moisture was measured by weight loss after drying of the samples at 105°C to a constant weight (23). Ash content was determined by drying in a muffle furnace (Ney VULCAN 3-550A, American) at 550°C for 24 h until ashes were white (23). Crude protein content was calculated by converting the nitrogen content (6.25 × N) determined by Kjeldahl’s method (23). Fat was determined by the Soxhlet extraction method (23).

### 2.7. Mineral element composition

Mineral determination according to Massimiliano et al. (24) with few modifications. 5 g of samples were digested in concentrated HNO_3_. The digest was quantitatively transferred to a 50 mL volumetric flask and brought up to volume with ultra-pure water. All minerals were determined by atomic absorption spectrometry (Agilent 7,900 ICP-MS, Perkin-Elmer, Santa Clara, CA, United States) against aqueous standards.

### 2.8. Fatty acid composition

Extraction of lipids: 2.0 g of minced fish was weighed into a centrifuge tube, 15 mL of chloroform-methanol (V/V = 2:1) solution containing 0.01% BHT was added, homogenized twice in an ice bath (10,000 r/min, 2 × 15 s, 30 s intervals), the volume was fixed to 30 mL, left to stand for 1 h and filtered. The filtrate was added to 0.2 times the volume of 0.85% saline and centrifuged at 3000 r/min for 10 min. The upper layer of water and impurities such as methanol were aspirated off and the lower layer of lipid liquid was transferred to a 50 mL pointed centrifuge tube and purged with nitrogen to concentrate the lipid.

Methylation of fatty acids: 2 mL of 14% trifluoromethylboron-methanol solution was added to the concentrated lipids and the methylation reaction was carried out in a water bath at 60°C for 30 min. After cooling to room temperature, 1 mL of hexane and 1 mL of distilled water were added and the solution was shaken for 1 ~ 2 min.

Fatty acid methyl esters (FAMEs) were analyzed by gas chromatography–mass spectrometry (GC–MS QP2100, Shimadzu, Tokyo, Japan) with an HP-5 MS capillary column (30 m × 0.25 mm × 0.25 μm; Palo Alto, CA, United States) as previously reported (25). The FAMEs were identified by comparing the relative retention time with that of authentic standards (37-component FAMEs mix; Supelco Inc., Bellefonte, PA, United States) and referenced with the NIST14 mass spectral database.

GC conditions: the carrier gas used was helium with a flow rate of 1.52 mL/min and a splitting ratio of 1:30; injection volume 1 μL, inlet temperature 230°C; ramp-up procedure: 110°C for 4 min, ramp-up to 160°C at 10°C/min and then hold for 1 min. The final ramp-up to 240°C was at 5°C/min and held for 15 min.

MS conditions: ion source temperature set to 200°C; solvent delay of 3 min; electron energy of 70 eV; mass scan range of 40–550 m/z.

### 2.9. Amino acid composition

The pre-treatments of amino acid samples were performed according to the method of Guo et al. (26). A total of 2 g of the sample was placed into a hydrolysis tube, to which hydrochloric acid (6 M, 15 mL) and 3–4 drops of phenol were added and fully mixed. The tubes were then filled with nitrogen gas for 3–4 times and closed, followed by hydrolysis in a thermoelectric thermostat drying box (DHG-9035A; Shanghai Jinghong Experimental Equipment Co., Ltd., China) at 110 ± 1°C for 24 h and then cooled to room temperature. After hydrolysis, the solution was transferred into a 50 mL volumetric flask, and diluted with water to a volume of 50 mL. The liquid was filtered, and 1 mL of the resulting solution was dried in a vacuum drying oven (DZF-6051; Shanghai Jinghong Experimental Equipment Co., Ltd., China), then rehydrated using 1 mL deionized water and dried again 2–3 times. The sample and 1.0 mL of sodium citrate buffer (pH = 2.2) were mixed and filtered by membrane filters (0.22 μm). Equal volumes of samples and mixed amino acid standard liquids were injected and analyzed by the high speed automatic amino acids analyzer (Hitachi, Model 835–50, Hitachi, Tokyo, Japan).

According to the FAO/WHO (1973) recommended amino acid scoring model and egg protein amino acid scoring model, amino acid score (AAS), chemical score (CS) and essential amino acid index (EAAI) were calculated as follows (27):


$$AAS=\frac{\begin{array}{l} \mathrm{Amino acid mass fraction of protein in sample}\;\mathrm{mg}\\ /g\;N \end{array}}{\begin{array}{l} \mathrm{The corresponding amino acid mass fraction in the}\;\mathrm{FAO}\\ /\mathrm{WHO}\;\mathrm{scoring standard mode}\;\mathrm{mg}/g\;N \end{array}}$$

$$CS=\frac{\begin{array}{l} \mathrm{Amino acid mass fraction of protein in sample}\;\mathrm{mg}\\ /g\;N \end{array}}{\mathrm{Amino acid mass fraction in}\;\mathrm{egg}\;\mathrm{protein}\;\mathrm{mg}/g\;N}$$

$$EAAI={\left(\frac{T_{Val}}{S_{Val}}\times \frac{T_{Leu}}{S_{Leu}}\times \cdots \times \frac{T_{Lys}}{S_{Lys}}\right)}^{\frac{1}{n}}\times 100$$

where, n is the number of comparative amino acids; *T* is the essential amino acid content of experimental protein; *S* is the essential amino acid content of egg protein.

### 2.10. Volatile flavor components

A total of 2.0 g of minced fish meat from each sample was placed in a 15 mL headspace vial containing 5 mL of saturated sodium chloride solution. Magnetic rotors were placed in the bottles and heated on a magnetic stirrer at 65°C for 10 min. The activated PDMS/DVB extraction head (65 μm) was used for headspace adsorption for 40 min. When the adsorption was completed, the extraction head was quickly inserted into the inlet of the gas chromatograph and desorbed for 10 min.

GC conditions: an Rtx®-WAX column (30 m × 0.25 mm, 0.25 μm) was used, the carrier gas was helium, the flow rate was 1.00 mL/min, the split ratio was 1:20, and the inlet temperature was 250°C. Temperature program: the initial temperature of the column was kept at 40°C for 2 min, after which the temperature was raised to 200°C at a rate of 6°C/min and kept at 200°C for 3 min. Finally, the temperature was heated to 250°C at a rate of 10°C/min and maintained for 3 min at the final temperature.

MS conditions: The ion source temperature was 230°C, the electron energy was 70 eV, and the mass scanning range was 35 ~ 350 m/z.

### 2.11. Sensory evaluation

The samples were randomly distributed among 20 trained panelists (10 females and 10 males), and quantitative descriptive analysis was performed. The evaluation was carried out separately by each evaluator, and communication was prohibited during the evaluation process. Each sample of approximately 5 g was placed in a clean cup with a random 3-digit code. Evaluators consumed at room temperature, once for each sample. The evaluator needed to gargle before each sample evaluation to rule out the effect of the previous sample. Color, appearance, flavor, taste and mouthfeel were used as descriptive indexes and are shown in Supplementary Table S1. Each index had a highest score of 10, and the average score from all the panelists was considered for the data analysis. All samples were evaluated once.

### 2.12. Statistical analysis

All experiments were conducted at least triplicate, and the data were analyzed using the statistical package SPSS 25.0 (SPSS, Chicago, IL, United States). Analysis of variance (ANOVA, *p* < 0.05) and Duncan’s multiple range tests were used to determine the significance of the difference between the samples. Bar graphs were generated using GraphPad Prism9 (Insightful Science Inc., California, MA, United States). Pie graphs were generated using Origin Pro (version 2021; Origin Lab Inc., Northampton, MA, United States). Cluster heat maps were generated using TBtools (Toolbox for Biologists, version 1.116, China). Correlation heat maps were visualized by R Studio-4.2.3 (R Studio Inc., Boston, MA, United States).

## 3. Results and discussion

### 3.1. Cooking loss and proximate composition

**Figure 1:**
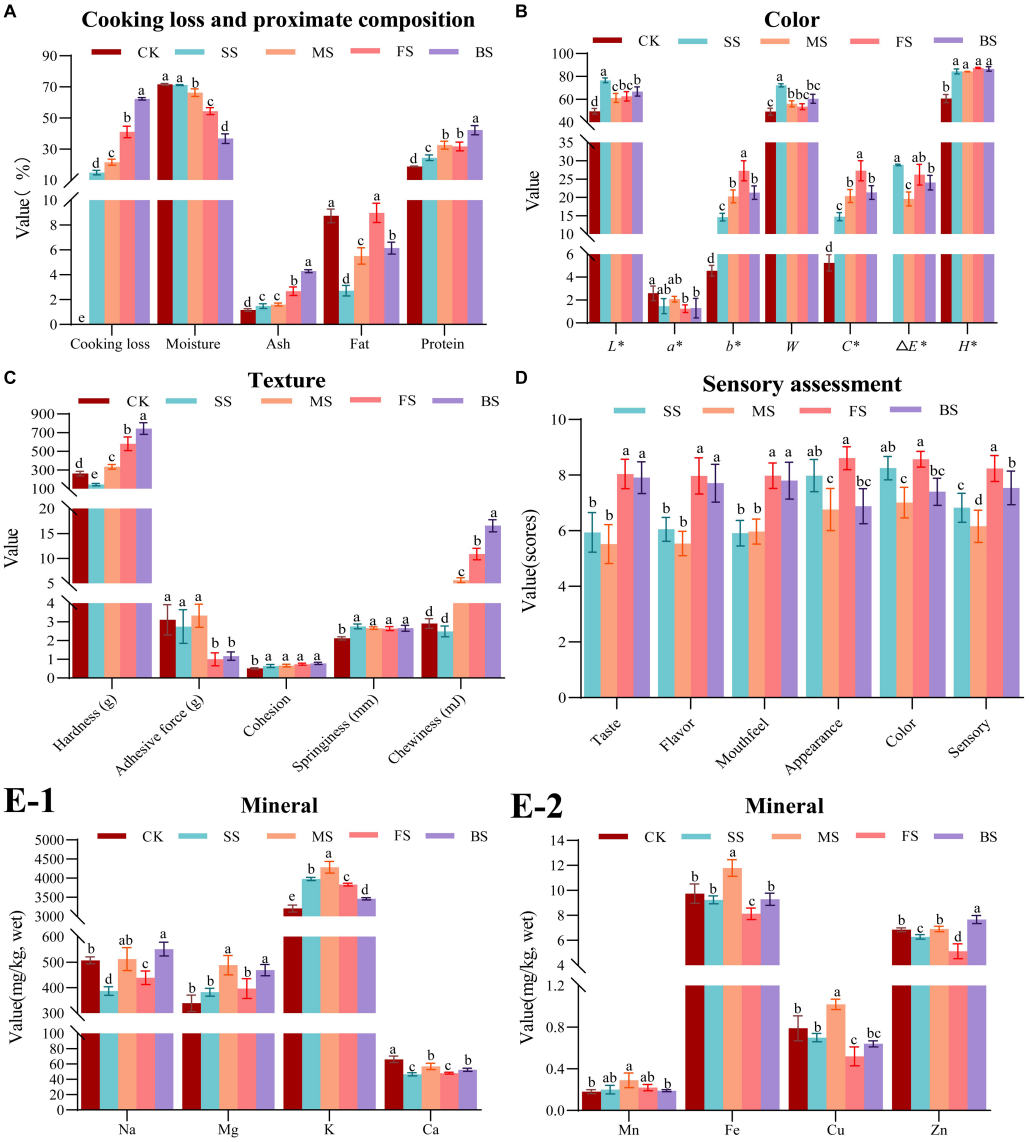
Effect of different cooking methods on edible quality in golden pomfret. **(A)** Cooking loss and proximate composition; **(B)** Color; **(C)** Texture; **(D)** Sensory assessment; **(E-1,2)** Mineral. Results are means ± standard error (*n* = 5). Different letters on the data indicate significant differences between groups (*p* < 0.05).

It is evident that the weights of all the samples decreased after cooking (Figure 1A). This can be attributed to the dissolution of soluble nitrogen, separation of fat, and loss of water during heat treatments. Among these factors, water loss was found to be the most significant. Furthermore, it is apparent that the moisture content of the four cooked samples had also decreased to varying degrees, which is consistent with a previous report (28). The rate of cooking loss is a crucial indicator of a muscle’s water holding capacity (WHC). Muscles contain bound water, free water, and immobilized water, with immobilized water being the primary factor in determining WHC. This type of water is located between myofibrils and myolemma, and its presence is dependent on the spatial structure of myofibrillar protein. If the protein is in a tight state, the network space is small, resulting in a lower WHC. Conversely, if the protein is in an expanded state, the network space is larger, leading to a greater WHC (29). The combination of photoelectricity and microwaves had a synergistic effect on the baked samples (BS), resulting in severe dehydration due to the damaged muscle structure. The BS had the lowest moisture content, which is 36.68%, significantly (*p* < 0.05) lower than the other four samples, followed by fried sample (FS) and microwaved sample (MS). However, due to the continuous supplementation of water vapor on the surface of the fish during heating, the steamed sample (SS) had the lowest cooking loss rate (14.81%) and highest moisture content (71.21%). There was no significant difference (*p* > 0.05) in moisture content between SS and control sample (CK, 71.65%), which indicating that steaming is the best cooking method for maintaining WHC among four cooking methods.

All cooking methods had a significant effect on the proximate compositions of golden pomfret. The most notable change was a decrease in moisture content, leading to a significant increase in protein and ash contents in the cooked samples. However, in our research, the fat content showed a slight increase only in FS but decreased under other cooking methods, which was consistent with the cooking results of Dong et al. (30) in turbot. This result caused by the high fat content in FS which was attributed to the evaporation of water accompanied by the infiltration of oil. While the decrease of fat content in other samples was due to the formation of small molecular substances from the heat reaction of fat, which dissolved in free water and was lost with it. Because water vapor can carry part of the fat of the fish, the relative content of the crude fat of the steamed fish was the lowest among samples cooked with the four methods, which was only 2.72%. The steaming method of fish processing is more in line with the needs of people on a low-fat diet.

### 3.2. Color

The concentration and chemical state of myoglobin, hemoglobin and cytochrome have a significant impact on the overall color of fish muscle (31). Temperature rose with all four cooking methods, which resulted in denaturation of myoglobin, Maillard reaction, destruction of pigments and oxidation of lipids to different degrees. As shown in Figure 1B, the L^*^, b^*^, C^*^, △E^*^ H^*^ and whiteness of cooked samples increased significantly, while a^*^ decreased significantly, which was in agreement with Thiansilakul et al. (32). Ocaño-Higuera et al. (33) reported that the higher the moisture content in muscle tissue, the higher the reflectivity of the muscle surface, and the higher the L^*^ value. In this study, steamed fish L^*^ (76.63) was higher than that of the other cooking methods, which could be partly attributed to its higher water content. In addition, the muscle with low moisture content was more likely to deposit pigments, which led to a decrease in muscle whiteness. Therefore, the whiteness of SS with high moisture content was higher. There was no significant difference (*p* > 0.05) in whiteness among MS, FS and BS, but the whiteness was significantly (*p* < 0.05) higher than CK. The main reason was a significant b^*^ increase led by lipid oxidation during heating (34). In this study, we found that the b^*^ and C^*^ of FS was the highest, while SS was the lowest. It was speculated that lipid oxidation was relatively stronger under high temperature frying conditions, and steaming could effectively inhibit lipid oxidation.

### 3.3. Texture

There were differences in the temperature, heat transfer rate, heat transfer medium and ripening mechanisms of steaming, microwaving, frying and baking methods, which had different effects on the textural characteristics of fish (Figure 1C). With the participation of aqueous medium, thermal processing converted collagen in the muscle connective tissue into gelatin, which softened the fish (35). Additionally, it can also make myofibrillar protein condense and contract, and muscle lose water and harden (36). The interaction between the two causes the difference in fish texture under different cooking methods. Except for SS whose hardness and chewiness were significantly lower than CK, the other groups were significantly increased. The hardness and chewiness of BS were the highest, which were 183.66 and 469.42% higher than CK, respectively. Next, the FS exhibited hardness and chewiness, which were 121.10 and 273.88% higher than CK, respectively. The order of hardness and chewiness among different cooking methods was consistent with that of the cooking weight loss rate but contrary to that of moisture content. Therefore, it is speculated that hardness and chewiness may be related to moisture content. The steamed, microwaved and fresh groups showed no significant difference in adhesion, while the FS and BS showed a significant decrease in adhesion compared with CK. The springiness and cohesion increased slightly after cooking, but there was no significant difference among the groups.

### 3.4. The sensory assessment

The scores of mouthfeel, taste and flavor of BS and FS were significantly higher than SS and MS (Figure 1D), which was consistent with the results of higher hardness and chewiness of fried and baked groups, higher UAAs and SAAs content of FS and BS in 3.7 and prominent aroma characteristics of pyrazine compounds of FS and BS in 3.8. The shape of the FS and SS was well preserved, and the appearance scores were relatively higher. The MS and BS were conducted in a microwave oven, and the moisture inside the fish meat was evaporated and expanded by the heat so that the surface of the fish meat was broken and cracked, the shape was broken and irregular, and the appearance score was lower. The SS were white and bright, and the FS were bright and an attractive yellow. The color of MS and BS was relatively dim and uneven. The highest sensory score was for the FS, followed by the BS and SS, and the lowest score was MS.

### 3.5. Mineral element composition

**Figure 2:**
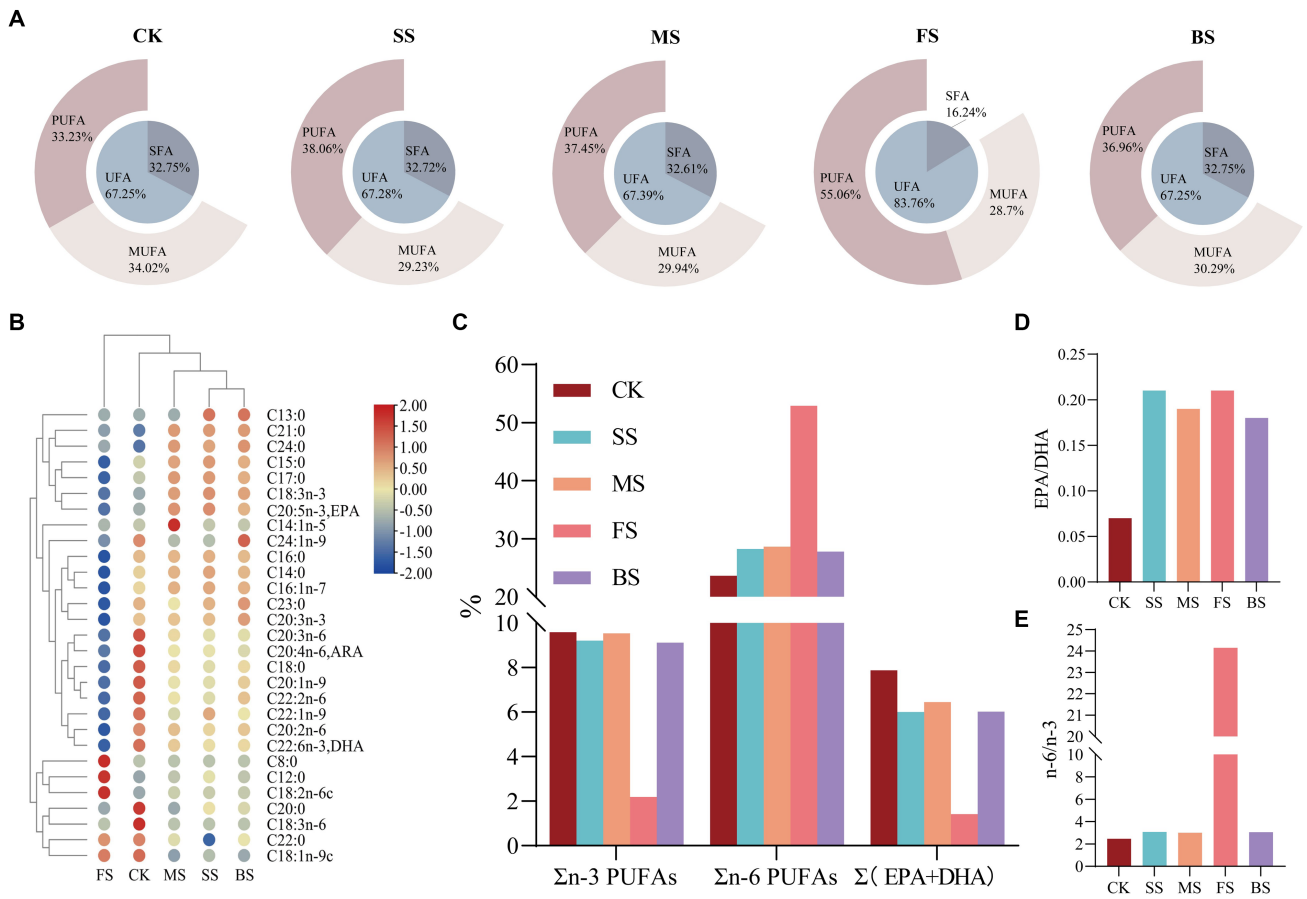
Effect of different cooking methods on fatty acids (FAs) composition in golden pomfret. **(A)** The relative contents of saturated fatty acids (SFAs), unsaturated fatty acids (UFAs), mono-unsaturated fatty acids (MUFAs) and poly-unsaturated fatty acids (PUFAs); **(B)** Heat map of the levels of FAs compounds; **(C)** n-3 PUFAs、n-6 PUFAs & (EPA + DHA).; **(D)** EPA/DHA; **(E)** n-6/n-3.

The Na content of the BS increased significantly, while FS and SS decreased significantly. There was no significant difference in Na content between CK and MS (Figure 1E-1), whereas Hosseini et al. (37) showed that boiling significantly reduced Na content in fish, while baking and frying had no significant effect on Na content in fish. In general, foods lacking Na taste bland, but a low sodium diet is more beneficial to human health. SS has the lowest Na content, which means that the same serving of golden pomfret with the steaming method has the lowest Na intake by the human body than other methods. This is more in line with a low sodium healthy diet. The changes in Mg and Mn content of all samples were insignificant (*p* > 0.05), except for the MS in which the Mg and Mn content increased significantly. This result is similar to the results reported (38). The K content of CK was found to be 3206.86 mg/kg. However, the K content of cooked fish increased significantly, which was consistent with the results of Ersoy et al. (39). In this study, Ca content was significantly decreased by the different cooking methods, while Badiani et al. (40) found that the Ca content of European sea bass decreased slightly after household cooking but not significantly. The contents of Cu and Fe increased significantly in MS, decreased significantly in the FS (Figure 1E-2). There was no significant difference in the contents of Cu and Fe in CK, SS and BS. The content of Zn increased significantly after baking, decreased significantly after frying and steaming, and there was no significant difference in the Zn content between CK and MS. In terms of the content of elements such as Na, Mg, K, Ca, Mn, Fe, Zn and Cu, BS and MS better preserved elemental compositions among the four cooking methods, while the losses of SS and FS were more notable. This result was due to the introduction of exogenous media oil and water in SS and FS, resulting in the loss of mineral elements with the juice during the cooking process.

### 3.6. Fatty acid composition

Heat treatment can lead to lipid oxidation of fish, so the fatty acid composition of fish under different cooking methods is very different from CK (Figure 2; Supplementary Table S2). The contents of saturated fatty acids (SFAs) and unsaturated fatty acids (UFAs) in SS, MS and BS were similar to CK. In details, the content of monounsaturated fatty acids (MUFAs) decreased slightly, while that of polyunsaturated fatty acids (PUFAs) increased slightly. The relative content of UFAs in FS (83.82%) was significantly higher than in the other groups (Figure 2A), among which the relative content of linoleic acid (18:2n-6) was as high as 52.50% (Supplementary Table S2). This was mainly due to the sunflower seed oil used for frying in this study. During the frying process, vegetable oil penetrates into the fish and causes changes to the fatty acid composition (41). The balance between the degradation and production of single fatty acids may be the reason of similar relative contents of the three types of fatty acids in SS, MS, BS and CK (42). There was a significant drop of arachidic acid (C20:0), γ-linolenic acid (C18:3n-6), dihomo-γ-linolenic acid (C20:3n-6), arachidonic acid (C20:4n-6, ARA) and docosahexaenoic acid (C22:6n-3, DHA) in CK. The significant rise of pentadecanoic acid (C15:0), heptadecanoic acid (C17:0), heneicosanoic acid (C21:0), tetracosanoic acid (C24:0), *ɑ*-linolenic acid (C18:3n-3) and eicosapentaenoic acid (C20:5n-3, EPA) in SS, MS and BS could also be clearly seen. The changes observed may be the consequences of the water loss and lipid oxidation produced by thermal processing. The increase of myristoleic acid (C14:1n-5) in MS and nervonic acid (C24:1n-9) in BS were also noteworthy.It is speculated that cooking at certain temperatures may contribute to the accumulation of these two PUFAs.

Long-chain PUFAs, such as ARA, EPA and DHA, have important physiological functions in brain development, infant intelligence and vision, and they are also regarded as vital evaluation indicators of nutritional value. The ARA and DHA content of golden pomfret were all significantly reduced after cooking, while the EPA content of cooked samples was significantly increased except for FS (Figure 2B). The loss of n-3 PUFAs in the FS was notable, whose EPA + DHA decreased from 7.88 to 1.42% (Figure 2C; Supplementary Table S2), while EPA + DHA in other groups were all above 6.00%. This is consistent with the results of Gladyshev et al. (43) in the study of several marine fish. Kitson et al. (44) found that salmon fried with artificial butter rich in EPA and DHA could prevent the loss of EPA and DHA, while when cooked with rapeseed oil or no oil, the content of EPA and DHA decreased significantly. The DHA + EPA of MS maintained stability, and the relative content was 6.45%. The EPA/DHA value increased after all cooking methods (Figure 2D).

The n-3 and n-6 fatty acids in PUFAs cannot be synthesized but are indispensable in the human body and must be obtained from the diet. The n-6/n-3 fatty acid ratio is a key factor in the synthetic balance of eicosanoids in the body and is of great significance in balancing diet and nutritional collocation. The excessive n-6/n-3 PUFA ratio in the diet promotes the development of cardiovascular disease, cancer and autoimmune diseases (45). The ratio of n-6/n-3 PUFAs in FS was as high as 24.15, which was 8–10 times that of other treatment groups (Figure 2E; Supplementary Table S2). Excessive intake is not conducive to cardiovascular health. SS, MS and BS were more suitable for consumers, and their dietary fatty acid ratios were more in accordance with the human health requirements proposed by FAO/WHO (SFAs:MUFAs:PUFAs = 1:1:1; n-6/n-3 = 5–10/1).

### 3.7. Amino acid composition

**Figure 3:**
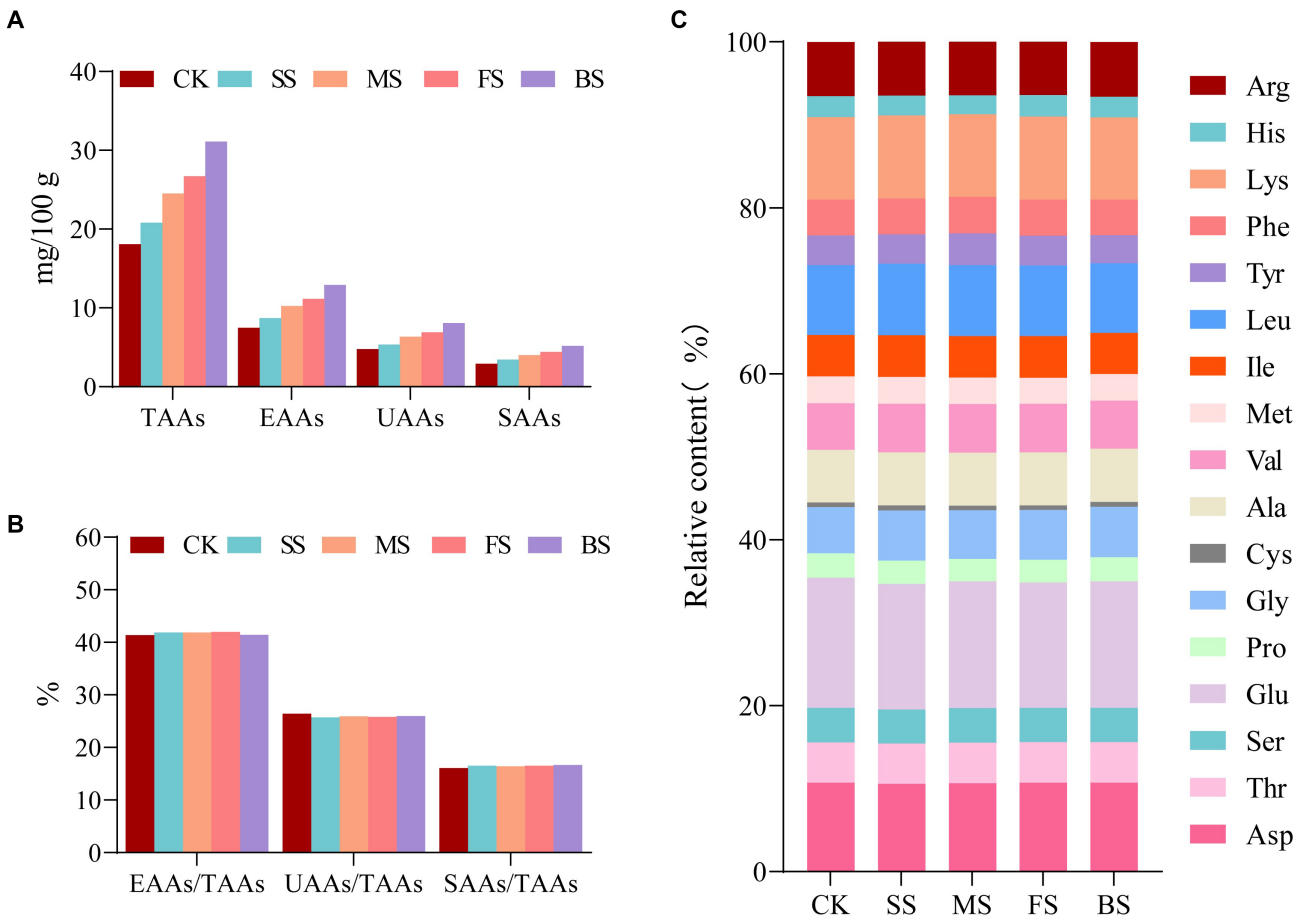
Effect of different cooking methods on amino acid composition in golden pomfret. **(A)** The contents of total amino acids (TAAs), essential amino acids (EAAs), umami amino acids (UAAs) and sweet amino acids (SAAs); **(B)** EAAs/TAAs, UAAs/TAAs and SAAs/TAAs; **(C)** The relative contents of amino acid composition.

As these four types of cooking methods resulted in a decrease in water content, an increase in protein content and protein degradation in the samples, the total amino acids (TAAs), essential amino acids (EAAs), umami amino acids (UAAs, Asp+Glu) and sweet amino acids (SAAs, Ser + Cly + Ala) increased after cooking in the order of BS > FS> MS> SS (Figure 3A). The difference between the EAAs/TAAs, UAAs/TAAs, SAAs/TAAs in CK and others was negligible (Figure 3B). UAAs and SAAs content in BS (8.08 mg/100 mg; 5.19 mg/100 mg) was much higher than FS (6.90 mg/100 mg;4.22 mg/100 mg), MS (6.35 mg/100 mg;4.02 mg/100 mg) and SS (5.35 mg/100 mg;3.44 mg/100 mg; Supplementary Table S3). These results reflect those of Wang (12) who also found that the roasted and fried scallops contained more sweet amino acids and umami amino acids than other samples. In general, cooking methods have little effect on the proportion of the amino acid composition but have a significant effect on the contents of each amino acid (Figure 3C; Supplementary Table S3).

The EAAs were evaluated by the recommended standard amino acid scoring method and the whole egg model of the FAO/WHO. After cooking treatment, the essential amino acid score (AAS) and chemical score (CS) showed small fluctuations (Table 1). According to the AAS and CS scores, the first limiting amino acid of all cooked samples were consistent CK, and all of them were sulfur-containing amino acids (Met+ Cys). The second limiting amino acid was Val when scored by AAS, while the second limiting amino acid was Phe + Tyr in SS, FS and BS, and Val in MS and CK when scored by CS. The lysine content of golden pomfret was very high, and both the AAS and CS scores in cooked samples were 1.82–1.85 times and 1.41–1.42 times the corresponding content of the FAO/WHO and the whole egg models, respectively. Therefore, an increase in golden pomfret intake can improve daily lysine deficiency caused by eating more cereals, and this fish can be further processed into high-quality emulsions and infant supplements. According to the whole egg model, the essential amino acid index (EAAI) was calculated. After hot processing, the EAAI had different degrees of growth except for BS. The EAAI value of MS was the highest (94.89), and the protein balance of these samples was also better, which is more conducive to human digestion and absorption.

**Table 1:** Evaluation of essential amino acid composition.

Essential amino acid	CK		SS		MS		FS		BS	
	AAS	CS	AAS	CS	AAS	CS	AAS	CS	AAS	CS
Val	1.12**	0.85**	1.17**	0.89	1.18**	0.89**	1.18**	0.89	1.17**	0.88
Met+Cys	1.08*	0.62*	1.11*	0.63*	1.08*	0.61*	1.06*	0.6*	1.08*	0.61*
Ile	1.24	0.94	1.25	0.94	1.24	0.94	1.26	0.95	1.24	0.93
Leu	1.19	0.98	1.22	1.01	1.22	1.00	1.22	1.00	1.20	0.99
Thr	1.21	1.05	1.21	1.04	1.22	1.05	1.23	1.05	1.22	1.05
Lys	1.83	1.41	1.84	1.42	1.83	1.41	1.85	1.42	1.82	1.41
Phe + Tyr	1.30	0.87	1.30	0.87**	1.35	0.91	1.31	0.88**	1.26	0.85**
EAAI	93.51		94.70		94.89		94.70		93.38	

EAAI, the essential amino acid index; *The first limiting amino acid; **The second limiting amino acid.

### 3.8. Volatile flavor component

**Figure 4:**
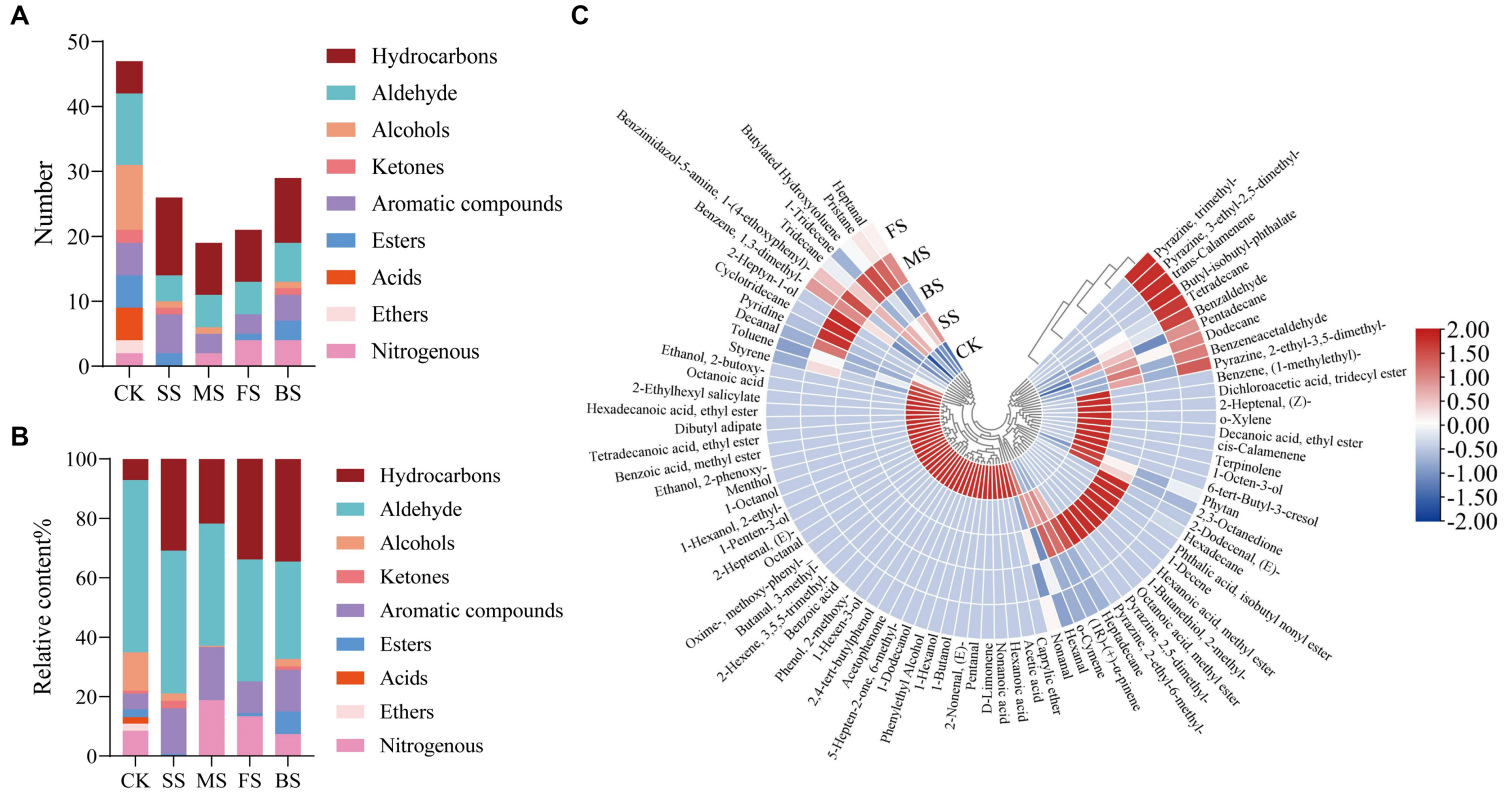
Effect of different cooking methods on volatile composition in golden pomfret. **(A)** Number of volatile composition; **(B)** Relative content of volatile composition; **(C)** Heat map of the levels of volatile compounds.

47, 26, 19, 21 and 29 volatile components were detected in CK, SS, FS, MS and BS (Figure 4A), respectively. Compared with CK, the number and relative content of hydrocarbons in cooked groups increased significantly (Figure 4B). Hydrocarbons are mainly derived from the homogeneous splitting of oxidative free radicals of fatty acid alkanes. C6-C19 alkanes have been identified in the volatiles of crustaceans and fish. However, hydrocarbon compounds have a higher threshold and contribute less to the direct flavor of fish but may contribute to the overall aroma of fish (46).

The aldehydes in fish are mainly derived from the oxidation and degradation of fatty acids, and the relatively low odor threshold of aldehydes contributes greatly to fish flavor (47). Although the relative content of aldehydes in golden pomfret decreased after cooking, it was still the most important flavor component of the fish. Hexanal and nonanal were detected in all samples, which were considered to be important related substances for fishy odor, and the relative contents were up to 36.30 and 11.23% in CK, respectively (Figure 4C; Supplementary Table S4). However, Hexanal lost after cooking, the relative contents were 32.85, 21.54, 11.83 and 9.07% in SS, MS, FS and BS. Nonanal also lost after cooking. Cooking may suppress the putrid odor which induced by high levels of hexanal and nonanal in fresh fish meat (48).

Alcohols are usually produced by the action of lipid oxidase and fatty acids, the decomposition of fatty acid secondary hydroperoxides or the reduction of carbonyl compounds (49). Four types of cooking methods resulted in a decrease in the type and relative content of alcohols, among which the volatile components of alcohols were not detected in FS. Ketones are mostly produced by thermal oxidation or amino acid degradation of UFAs, with unique fragrance and fruit aroma. The relative content of ketones in SS was significantly higher than others. Aromatic compounds increased in cooked groups, but acids and ethers disappeared after cooking. BS had the highest proportion of esters than others, with a high content of octanoic acid, methyl ester of 5.76%. Nitrogen-containing compounds originate from the Maillard reaction and thermal degradation of amino acids and thiamine. Most of them exist at very low concentrations in meat, but because of their low threshold, they are the most important flavor-causing substances in meat. Most of them have meat flavor, roast meat flavor, char flavor and nut flavor. The aroma characteristics of the pyrazine compounds in the FS and BS were outstanding. Each of these compounds contained three types of pyrazine nitrogen-containing compounds, and both contained 2-ethyl-3,5-dimethylpyrazine.

Generally, steaming and microwaving preserved the fragrance of the fish itself better, while frying and baking added the flavor of scorching and roasting. The results of this experiment are different from other fish flavor experiments (50), which may be due to the fish species, cultural environment, extraction methods, and instrument performance. The specific causes need to be further explored.

### 3.9. Correlation analysis

**Figure 5:**
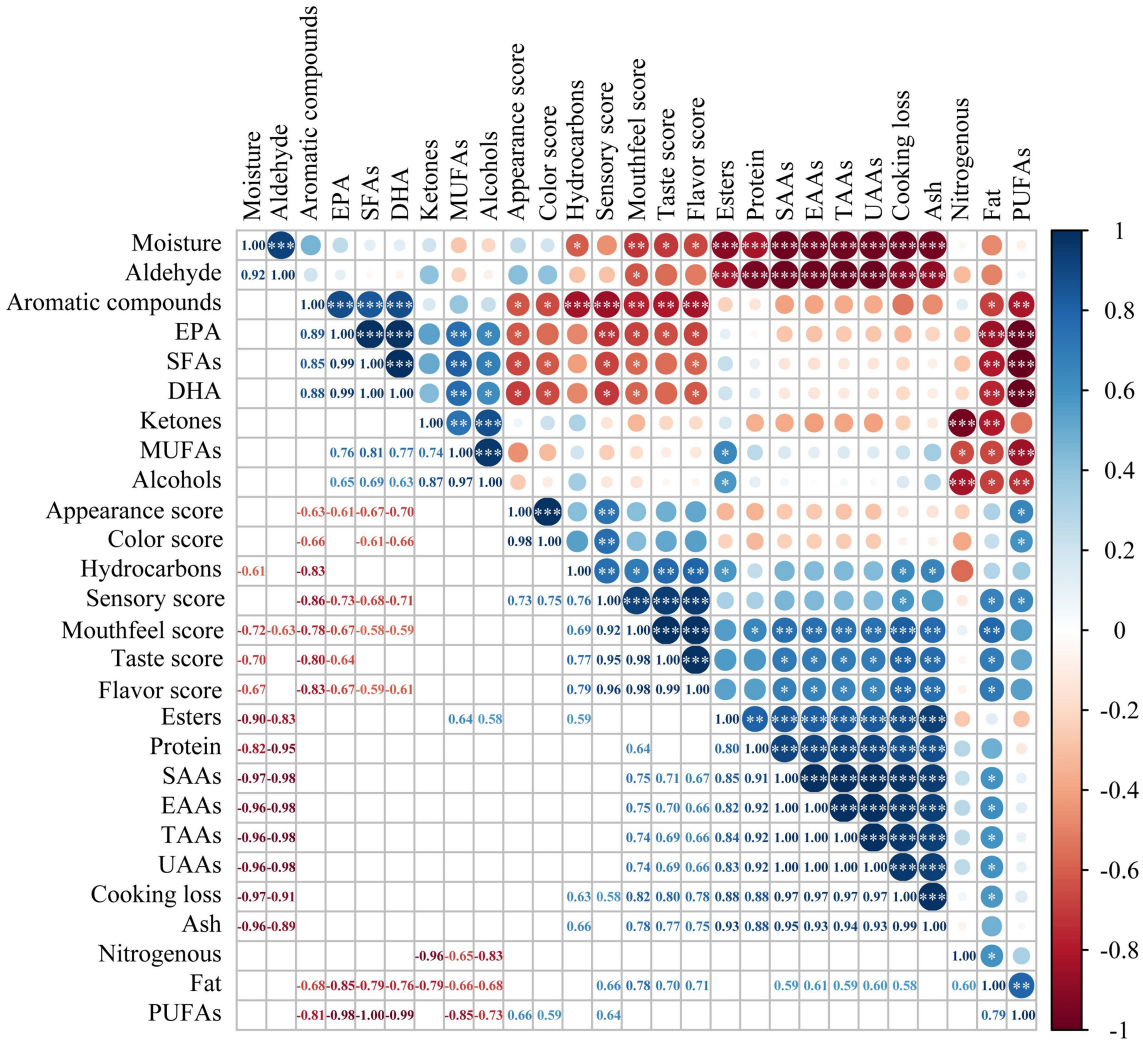
Correlation analysis of main indicators. *, ** and *** indicate a significant difference at the level of *p* < 0.05, *p* < 0.01 and *p* < 0.001, respectively.

The correlation between key indicators of the flavor, nutrition and edible quality during different cooking methods was analyzed (Figure 5). There was a significant negative correlation between moisture content and cooking loss, ash, protein, TAAs, EAAs, UAAs, SAAs, esters, hydrocarbons content and mouthfeel, taste, flavor scores. According to the previous analysis, the cooking losses of the different cooking methods resulted in moisture loss of the golden pomfret filets. The moisture loss caused the aggregation of flavor substances and nutrients, which could explain the above correlation. The PUFAs content significantly positively correlated with fat content, appearance, color and sensory scores. The PUFAs content significantly negatively correlated with the SFAs, DHA, EPA, MUFAs, aromatic compounds and alcohol contents. The addition of exogenous oils and fat oxidation caused by cooking resulted in changes in fatty acid composition. It affected the appearance, color, nutrition and flavor of golden pomfret. A correlation between fatty acid oxidation and the odor of pomfret was also found by Wang et al. (6) in the study of fermented golden pomfret. It was clear from the above that there was a close correlation between nutrition and flavor in different cooking methods, which together affected the edible quality of golden pomfret. During thermal processing, the increase of cooking loss and the change in FAs and flavor brought changes in the overall sensory score. Sensory scores were positively correlated with cooking loss, fat, PUFAs, hydrocarbons content and negatively correlated with SFAs, aromatic compounds content. Therefore, cooking loss and fat content can be used as simple evaluation indicators to compare the overall quality of different cooking methods.

## 4. Conclusion

Because of cooking loss, fat oxidation and the addition of exogenous oils, different cooking methods (steaming, frying, microwaving and baking) had significant effects on nutrition, flavor and edible quality of golden pomfret. SS had the lowest cooking loss, the highest moisture content, the lowest fat content, white and bright color, complete appearance, and the fresh flavor of the fish itself was better preserved. MS was dim and uneven, with the least number of flavor types detected and the lowest total sensory score. However, this cooking method better preserved mineral elements, DHA + EPA and other nutrients. FS was bright and yellow color, with the prominent aroma characteristic of pyrazine compounds and the highest total score from the sensory evaluation. But the ratio of n-6/n-3 PUFAs in FS was too high and harmful to cardiovascular health. BS had the highest cooking loss, the lowest moisture content and the highest hardness, chewiness, UAAs and SAAs content, but the protein balance and digestibility were possibly lower. Steaming and microwaving treatment of golden pomfret resulted in better nutritional preservation, which was more conducive to human health. Frying and baking treatment of golden pomfret had better taste, flavor and higher sensory scores. In actual production, people can choose the appropriate cooking methods or comprehensively use a variety of cooking methods for fish processing according to their own needs.

According to the indicators of this experiment, the flavor, nutrition and edible quality of golden pomfret under different cooking methods were related and interactive. Cooking loss and fat content can be used as simple evaluation indicators to compare the overall quality of different cooking methods. The above indicators can also be used in practical industrial production as indicators for judging the end of cooking to improve production efficiency.

## Data availability statement

The original contributions presented in the study are included in the article/Supplementary material, further inquiries can be directed to the corresponding author.

## Author contributions

TX: experiments, statistical analysis of data, visualization, and writing–original draft. XM: project support, research methodology, and conceptualization. YW: project proposal, experimental design, review and editing. LW, JS, YS, and SC: review and editing. FC, XC, and CF: experiments. All authors contributed to the article and approved the submitted version.

## Funding

This work was supported by the earmarked fund for Marine Fish Culture Industry (CARS-47) and the Young Top-notch Talent Cultivation Program of Hubei Province.

## Conflict of interest

The authors declare that the research was conducted in the absence of any commercial or financial relationships that could be construed as a potential conflict of interest.

## Publisher’s note

All claims expressed in this article are solely those of the authors and do not necessarily represent those of their affiliated organizations, or those of the publisher, the editors and the reviewers. Any product that may be evaluated in this article, or claim that may be made by its manufacturer, is not guaranteed or endorsed by the publisher.
