# Supplementary material for: Insights into nutrition, flavor and edible quality changes of golden pomfret (Trachinotus ovatus) fillets prepared by different cooking methods
Source: Front Nutr. 2023 Jul 7;10:1227928. doi: 10.3389/fnut.2023.1227928 (PMC10361837; doi:10.3389/fnut.2023.1227928)
Supplement: Supplementary file 1 [file Data_Sheet_1.docx]

Supplementary Material

Insights into nutrition, flavor and edible quality changes of golden pomfret (*Trachinotus ovatus*) fillets prepared by different cooking methods

Tian Xiong^1, 2^, Xin Mei^1, *^, Yanyan Wu^2, *^, Lan Wang^1^, Jianbin Shi^1^, Yong Sui^1^, Sha Cai^1^, Fang Cai^1^, Xueling Chen^1^, Chuanhui Fan^1^

*** Correspondence:**

*Xin Mei

E-mail: meixin0898@163.com

*Yanyan Wu

E-mail: wuyygd@163.com

# Supplementary Tables

**Table S1. Criterions of sensory evaluation**

| Indexes | Scores | Description |
| --- | --- | --- |
| Taste (20%) | 8~10 | Delicious and rich in fish taste |
|  | 5~7 | Lighter in fish taste but acceptable |
|  | 1~4 | Unpleasant taste or no fish taste |
| Flavour (20%) | 8~10 | Strong flavor of fresh and fragrance instead of bad smell of fish |
|  | 5~7 | Light fresh flavor and almost no bad smell of fish |
|  | 1~4 | No fresh flavor but heavy bad smell of fish |
| Mouthfeel (20%) | 8~10 | Tender and soft, with moderate hardness and good chewiness. |
|  | 5~7 | General elasticity and chewiness |
|  | 1~4 | Too loose to chew or too dry and hard to chew |
| Appearance (20%) | 8~10 | Complete and regularity appearance |
|  | 5~7 | Basically complete and acceptable appearance |
|  | 1~4 | Seriously damaged and irregularity appearance |
| Color (20%) | 8~10 | Attractive and glossy color with (amber or bright white). |
|  | 5~7 | Color with slightly glossy(yellow or white but not bright) |
|  | 1~4 | Dull or uneven color (dark brown or gray white) |

**Table S2. Fatty acid composition of samples（%）**

| Fatty acid | CK | SS | MS | FS | BS |
| --- | --- | --- | --- | --- | --- |
| C8:0 | - | - | - | 0.01 | - |
| C12:0 | 0.02 | 0.04 | 0.03 | 0.09 | 0.03 |
| C13:0 | - | 0.01 | - | - | 0.01 |
| C14:0 | 1.72 | 2.00 | 1.91 | 0.39 | 1.88 |
| C15:0 | 0.20 | 0.30 | 0.29 | 0.06 | 0.28 |
| C16:0 | 22.60 | 23.10 | 22.80 | 11.00 | 22.60 |
| C17:0 | 0.24 | 0.36 | 0.36 | 0.11 | 0.34 |
| C18:0 | 6.76 | 4.98 | 5.27 | 3.31 | 5.28 |
| C20:0 | 0.52 | 0.38 | 0.31 | 0.31 | 0.36 |
| C21:0 | - | 0.05 | 0.05 | 0.01 | 0.05 |
| C22:0 | 0.55 | - | 0.32 | 0.52 | 0.34 |
| C23:0 | 0.13 | 0.13 | 0.11 | 0.04 | 0.14 |
| C24:0 | - | 1.29 | 1.36 | 0.40 | 1.40 |
| C14:1n-5 | 0.02 | 0.02 | 0.21 | - | 0.02 |
| C16:1n-7 | 2.43 | 2.84 | 2.77 | 0.58 | 2.76 |
| C18:1n-9c | 27.90 | 25.00 | 24.40 | 27.60 | 24.60 |
| C20:1n-9 | 2.06 | 1.19 | 1.23 | 0.39 | 1.40 |
| C22:1n-9 | 0.75 | 0.62 | 0.42 | 0.09 | 0.47 |
| C24:1n-9 | 0.85 | 0.29 | 0.28 | 0.07 | 1.01 |
| C18:2n-6c | 20.50 | 26.20 | 26.40 | 52.50 | 25.60 |
| C18:3n-6 | 0.12 | - | - | - | - |
| C18:3n-3 | 1.31 | 2.80 | 2.68 | 0.71 | 2.64 |
| C20:2n-6 | 1.93 | 1.49 | 1.62 | 0.28 | 1.58 |
| C20:3n-6 | 0.15 | 0.08 | 0.09 | 0.02 | 0.08 |
| C20:4n-6 | 0.55 | 0.29 | 0.30 | 0.08 | 0.27 |
| C20:3n-3 | 0.40 | 0.41 | 0.40 | 0.06 | 0.46 |
| C20:5n-3 (EPA) | 0.50 | 1.03 | 1.01 | 0.25 | 0.91 |
| C22:2n-6 | 0.38 | 0.21 | 0.23 | 0.04 | 0.27 |
| C22:6n-3 (DHA) | 7.38 | 4.97 | 5.44 | 1.17 | 5.11 |
| ΣSFA | 32.74 | 32.63 | 32.81 | 16.24 | 32.71 |
| ΣMUFA | 34.01 | 29.96 | 29.31 | 28.72 | 30.26 |
| ΣPUFA | 33.22 | 37.47 | 38.17 | 55.10 | 36.92 |
| ΣUFA | 67.23 | 67.43 | 67.48 | 83.82 | 67.17 |
| Σn-3 PUFA | 9.59 | 9.21 | 9.53 | 2.19 | 9.12 |
| Σn-6 PUFA | 23.63 | 28.26 | 28.64 | 52.91 | 27.80 |
| n-6/n-3 | 2.46 | 3.07 | 3.00 | 24.15 | 3.05 |
| EPA/DHA | 0.07 | 0.21 | 0.19 | 0.21 | 0.18 |
| EPA+DHA | 7.88 | 6.00 | 6.45 | 1.42 | 6.02 |

*-, indicates not detected; C8:0, octanoic acid; C12:0, lauric acid; C13:0, tridecanoic acid; C14:0, myristic acid; C15:0, pentadecanoic acid; C16:0, palmitic acid; C17:0, heptadecanoic acid; C18:0, stearic acid; C20:0, arachidic acid; C21:0, heneicosanoic acid; C22:0, behenic acid; C23:0, tricosanoic acid; C14:1n-5, myristoleic acid; C24:0, tetracosanoic acid; C16:1n-7, palmitic acid; C18:1n-9c, oleic acid; C20:1n-9,eicosaenoic acid; C22:1n-9, erucic acid; C24:1n-9, nervonic acid; C18:2n-6c, linoleic acid; C18:3n-6, γ-linolenic acid; C18:3n-3, ɑ-linolenic acid; C20:2n-6, eicosadienoic acid; C20:3n-6, cis-8,11,14-Eicosatrienoic acid; C20:4n-6(ARA), Arachidonic acid; C20:3n-3, cis-11,14,17-eicosatrienoic acid; C20:5n-3 (EPA), cis-5,8,11,14,14-eicosapentaenoic; C22:2n-6, cis-13,16-docosadienoic acid; , C22:6n-3 (DHA), cis-4,7,10,13,16,19-docosahexaenoic; ΣSFA, total amount of saturated fatty acids; ΣMUFA, total amount of monounsaturated fatty acids; ΣPUFA, total amount of polyunsaturated fatty acids; ΣUFA, total amount of unsaturated fatty acids; Σn-3 PUFA, total amount of n-3 fatty acids; Σn-6 PUFA, total amount of n-6 fatty acids.*

**Table S3. Amino acid composition of samples（mg/100 mg sample）**

| Amino acids | CK | SS | MS | FS | BS |
| --- | --- | --- | --- | --- | --- |
| Asp | 1.94 | 2.20 | 2.61 | 2.86 | 3.34 |
| Thr | 0.88 | 1.01 | 1.20 | 1.31 | 1.52 |
| Ser | 0.75 | 0.85 | 1.02 | 1.10 | 1.29 |
| Glu | 2.84 | 3.15 | 3.74 | 4.04 | 4.74 |
| Pro | 0.53 | 0.58 | 0.66 | 0.73 | 0.90 |
| Gly | 1.01 | 1.26 | 1.44 | 1.61 | 1.90 |
| Cys | 0.10 | 0.13 | 0.14 | 0.15 | 0.18 |
| Ala | 1.15 | 1.33 | 1.56 | 1.71 | 2.00 |
| Val | 1.01 | 1.21 | 1.43 | 1.56 | 1.80 |
| Met | 0.59 | 0.68 | 0.79 | 0.84 | 1.00 |
| Ile | 0.90 | 1.04 | 1.22 | 1.34 | 1.54 |
| Leu | 1.52 | 1.79 | 2.10 | 2.28 | 2.62 |
| Tyr | 0.65 | 0.74 | 0.93 | 0.96 | 1.05 |
| Phe | 0.78 | 0.90 | 1.08 | 1.16 | 1.33 |
| Lys | 1.80 | 2.08 | 2.44 | 2.67 | 3.09 |
| His | 0.46 | 0.50 | 0.56 | 0.70 | 0.77 |
| Arg | 1.18 | 1.35 | 1.58 | 1.71 | 2.06 |
| TAAs | 18.09 | 20.80 | 24.50 | 26.73 | 31.13 |
| EAAs | 7.48 | 8.71 | 10.26 | 11.16 | 12.90 |
| UAAs | 4.78 | 5.35 | 6.35 | 6.90 | 8.08 |
| SAAs | 2.91 | 3.44 | 4.02 | 4.42 | 5.19 |
| EAAs/TAAs | 41.35% | 41.88% | 41.88% | 41.75% | 41.44% |
| UAAs/TAAs | 26.42% | 25.72% | 25.92% | 25.81% | 25.96% |
| SAAs/TAAs | 16.09% | 16.54% | 16.41% | 16.54% | 16.67% |

*Asp, aspartic acid; Thr, threonine; Ser, serine; Glu, glutamic acid; Pro, proline; Gly, glycine; Cys, cysteine; Ala, alanine; Val, valine; Met, methionine; Ile, isoleucine; Leu, leucine; Tyr, tyrosine; Phe, phenylalanine; Lys, lysine; His, histidine; Arg, arginine; TAAs,total amino acids; EAAs,essential amino acids(Thr+ Val+ Met + Ile + Leu + Phe + Lys ); UAAs, umami amino acids(Asp + Glu); SAAs, sweet amino acids(Ser + Cly + Ala).*

**Table S4. Volatile components of samples（mg/100 mg sample）**

|  | CK | SS | MS | FS | BS |
| --- | --- | --- | --- | --- | --- |
| Hydrocarbons | 7.07 （5） | 30.87 （12） | 21.80 （8） | 33.85 （8） | 34.50 （10） |
| D-Limonene | 0.39 | - | - | - | - |
| Dodecane | 0.78 | 5.64 | 9.47 | 14.73 | 13.60 |
| Cyclotridecane | - | - | 0.90 | - | - |
| Tridecane | - | 1.19 | 1.05 | 0.78 | 1.14 |
| Tetradecane | 0.49 | 1.05 | 1.72 | 6.43 | 2.63 |
| Pentadecane | - | 5.85 | - | 6.66 | 5.48 |
| Hexadecane | - | 0.11 | - | 0.18 | 2.51 |
| Heptadecane | - | 0.41 | - | - | 0.66 |
| 1-Tridecene | - | 1.95 | 3.11 | - | - |
| 1-Decene | - | - | - | - | 3.01 |
| Styrene | 4.94 | - | 2.23 | - | 0.66 |
| cis-Calamenene | - | 0.28 | - | - | - |
| trans-Calamenene | - | - | - | 0.37 | - |
| Pristane | - | 1.60 | 2.57 | 1.38 | - |
| 2-Hexene, 3,5,5-trimethyl- | 0.46 | - | - | - | - |
| (1R)-(+)-α-pinene | - | 0.50 | - | - | 0.56 |
| Phytan | - | 10.06 | 0.76 | 3.30 | 4.25 |
| Terpinolene | - | 2.24 | - | - | - |
| Aldehyde | 58.03 （11） | 48.09（4） | 41.08 （5） | 41.04 （5） | 32.82 （6） |
| Butanal, 3-methyl- | 1.89 | - | - | - | - |
| Pentanal | 0.39 | - | - | - | - |
| Hexanal | 36.30 | 32.85 | 21.54 | 11.83 | 9.07 |
| Heptanal | 0.87 | 8.73 | 9.00 | 6.11 | 3.23 |
| Octanal | 2.49 | - | - | - | - |
| 2-Heptenal, (E)- | 0.56 | - | - | - | - |
| 2-Heptenal, (Z)- | - | 1.06 | - | - | - |
| Nonanal* | 11.23 | 5.45 | 4.97 | 7.72 | 7.76 |
| 2-Dodecenal, (E)- | 0.29 | - | - | - | 3.25 |
| Decanal | 2.14 | - | 2.28 | - | - |
| Benzaldehyde | 1.55 | - | 3.29 | 10.91 | 4.95 |
| Benzeneacetaldehyde | - | - | - | 4.47 | 4.55 |
| 2-Nonenal, (E)- | 0.31 | - | - | - | - |
| Alcohols | 12.89 （10） | 2.45 （1） | 0.49 （1） | 0.00 （0） | 2.51 （1） |
| 1-Butanol | 0.89 | - | - | - | - |
| 1-Penten-3-ol | 0.91 | - | - | - | - |
| 1-Butanethiol, 2-methyl- | - | - | - | - | 2.51 |
| 1-Hexanol | 0.45 | - | - | - | - |
| 1-Hexen-3-ol | 0.94 | - | - | - | - |
| 2-Heptyn-1-ol | - | - | 0.49 | - | - |
| 1-Octen-3-ol | - | 2.45 | - | - | - |
| 1-Hexanol, 2-ethyl- | 6.67 | - | - | - | - |
| 1-Octanol | 1.43 | - | - | - | - |
| Menthol | 0.53 | - | - | - | - |
| Phenylethyl Alcohol | 0.44 | - | - | - | - |
| 1-Dodecanol | 0.16 | - | - | - | - |
| Ethanol, 2-phenoxy- | 0.48 | - | - | - | - |
| Ketones | 0.99 （2） | 2.52 （1） | 0.00（0） | 0.00 （0） | 1.18 （1） |
| 5-Hepten-2-one, 6-methyl- | 0.59 | - | - | - | - |
| 2,3-Octanedione | - | 2.52 | - | - | 1.18 |
| Acetophenone | 0.41 | - | - | - | - |
| Aromatic compounds | 5.32 （5） | 15.38 （6） | 17.80 （3） | 10.68 （3） | 13.96 （4） |
| Toluene | 2.23 | 1.17 | 1.25 | 0.71 | 0.84 |
| o-Cymene | 0.19 | 3.83 | - | - | 5.10 |
| Benzene, (1-methylethyl)- | - | 0.67 | - | - | - |
| Phenol, 2-methoxy- | 0.76 | - | - | - | - |
| 6-tert-Butyl-3-cresol | - | 0.20 | - | - | - |
| o-Xylene | - | 2.24 | - | - | - |
| Benzene, 1,3-dimethyl- | - | - | 2.97 | 2.79 | 1.97 |
| Butylated Hydroxytoluene | 1.82 | 7.27 | 13.58 | 7.17 | 6.06 |
| 2,4-tert-butylphenol | 0.31 | - | - | - | - |
| Esters | 2.61 （5） | 0.69 （2） | 0.00 （0） | 1.08 （1） | 7.68 （3） |
| Decanoic acid, ethyl ester | - | 0.20 | - | - | - |
| Butyl-isobutyl-phthalate | - | - | - | 1.08 | - |
| Hexanoic acid, methyl ester | - | - | - | - | 0.88 |
| Octanoic acid, methyl ester | - | - | - | - | 5.76 |
| Benzoic acid, methyl ester | 0.14 | - | - | - | - |
| Tetradecanoic acid, ethyl ester | 0.35 | - | - | - | - |
| Dibutyl adipate | 0.65 | - | - | - | - |
| Phthalic acid, isobutyl nonyl ester | - | - | - | - | 1.04 |
| Hexadecanoic acid, ethyl ester | 1.13 | - | - | - | - |
| 2-Ethylhexyl salicylate | 0.34 | - | - | - | - |
| Dichloroacetic acid, tridecyl ester | - | 0.50 | - | - | - |
| Acids | 2.18 （5） | 0.00 （0） | 0.00 （0） | 0.00 （0） | 0.00 （0） |
| Acetic acid | 1.02 | - | - | - | - |
| Hexanoic acid | 0.39 | - | - | - | - |
| Octanoic acid | 0.29 | - | - | - | - |
| Nonanoic acid | 0.22 | - | - | - | - |
| Benzoic acid | 0.26 | - | - | - | - |
| Ethers | 2.37 （2） | 0.00 （0） | 0.00 （0） | 0.00（0） | 0.00 （0） |
| Ethanol, 2-butoxy- | 1.42 | - | - | - | - |
| Octane, 1,1'-oxybis- | 0.95 | - | - | - | - |
| Nitrogenous | 8.52 （2） | 0.00 （0） | 18.83 （2） | 13.35 （4） | 7.34 （4） |
| Pyridine | 2.74 | - | 12.28 | - | - |
| Oxime-, methoxy-phenyl-_ | 5.79 | - | - | - | - |
| Pyrazine, trimethyl- | - | - | - | 6.39 | - |
| Pyrazine, 2-ethyl-3,5-dimethyl- | - | - | - | 2.19 | 1.54 |
| Pyrazine, 2,5-dimethyl- | - | - | - | - | 3.73 |
| Pyrazine, 2-ethyl-6-methyl- | - | - | - | - | 1.36 |
| Pyrazine, 3-ethyl-2,5-dimethyl- | - | - | - | 1.04 | - |
| Benzimidazol-5-amine, 1-(4-ethoxyphenyl)- | - | - | 6.55 | 3.74 | 0.70 |

Note:

- Indicates not detected
